# Delivery cost analysis of a reactive mass cholera vaccination campaign: a case study of Shanchol™ vaccine use in Lake Chilwa, Malawi

**DOI:** 10.1186/s12879-017-2885-8

**Published:** 2017-12-19

**Authors:** Patrick G. Ilboudo, Jean-Bernard Le Gargasson

**Affiliations:** 1 Agence de Médecine Préventive, Abidjan, Côte d’Ivoire; 2 Agence de Médecine Préventive, Ferney-Voltaire, France

**Keywords:** Cholera, Shanchol, Delivery costs, Choltool, Malawi

## Abstract

**Background:**

Cholera is a diarrheal disease that produces rapid dehydration. The infection is a significant cause of mortality and morbidity. Oral cholera vaccine (OCV) has been propagated for the prevention of cholera. Evidence on OCV delivery cost is insufficient in the African context. This study aims to analyze Shanchol vaccine delivery costs, focusing on the vaccination campaign in response of a cholera outbreak in Lake Chilwa, Malawi.

**Methods:**

The vaccination campaign was implemented in two rounds in February and March 2016. Structured questionnaires were used to collect costs incurred for each vaccination related activity, including vaccine procurement and shipment, training, microplanning, sensitization, social mobilization and vaccination rounds. Costs collected, including financial and economic costs were analyzed using Choltool, a standardized cholera cost calculator.

**Results:**

In total, 67,240 persons received two complete doses of the vaccine. Vaccine coverage was higher in the first round than in the second. The two-dose coverage measured with the immunization card was estimated at 58%. The total financial cost incurred in implementing the campaign was US$480275 while the economic cost was US$588637. The total financial and economic costs per fully vaccinated person were US$7.14 and US$8.75, respectively, with delivery costs amounting to US$1.94 and US$3.55, respectively. Vaccine procurement and shipment accounted respectively for 73% and 59% of total financial and economic costs of the total vaccination campaign costs while the incurred personnel cost accounted for 13% and 29% of total financial and economic costs. Cost for delivering a single dose of Shanchol was estimated at US$0.97.

**Conclusion:**

This study provides new evidence on economic and financial costs of a reactive campaign implemented by international partners in collaboration with MoH. It shows that involvement of international partners’ personnel may represent a substantial share of campaign’s costs, affecting unit and vaccine delivery costs.

**Electronic supplementary material:**

The online version of this article (10.1186/s12879-017-2885-8) contains supplementary material, which is available to authorized users.

## Introduction

Cholera is a diarrheal disease that produces rapid dehydration. Caused by ingestion of toxigenic *Vibrio cholerae* [1], the infection represents a serious public health problem in the developing world. The disease is a significant cause of mortality and morbidity, particularly for children and vulnerable people. The provision of safe water coupled with adequate hygiene and sanitation have been acknowledged as measures for the prevention of cholera [2, 3] and agreed upon by the international community through their adoption in the Sustainable Development Goals [4]. In spite of this, 2.9 million cases of cholera continue to occur worldwide every year with an estimated annual mortality of 95,000 [5]. A larger part of these deaths occur in sub-Saharan Africa where access to an improved drinking-water source remain problematic for thousands of people [6].

It has been largely accepted that improvement in water and hygiene conditions is unlikely to occur in the near future due to suboptimal funding and infrastructure. In recognition of this, OCVs have been recommended for cholera prevention by the World Health Organization (WHO) [1]. Consequently, Dukoral, and more recently Shanchol, a low-cost cholera vaccine, were prequalified for the prevention of cholera [7]. There is an abundance of literature demonstrating the protective efficacy of Shanchol vaccine [8–12]. As a consequence, there has been an increased enthusiasm in its use for the prevention of cholera. A review from the WHO reported that up to 35 OCV campaigns were conducted between July 2013 to July 2016 in response to emergency and endemic contexts in various settings [13].

Although Shanchol has been largely propagated as inexpensive, ongoing questions regarding its delivery costs exist. High vaccine delivery costs may discourage its use in resource-limited settings. However, evidence on Shanchol delivery costs is questionable due to inconsistent collection and reporting of cost data [14]. This study aims to provide a detailed analysis of Shanchol delivery cost focusing on the case study of a reactive vaccination campaign in Lake Chilwa, Malawi.

## Methods

### Study setting

The campaign was implemented in three administrative districts, including Machinga, Phalombe, and Zomba. In 2016, all three districts had an estimated total population of 1,895,625 inhabitants [15]. The districts’ economies are driven by agriculture and fishing. Health services are mainly delivered to these communities through health posts, clinics, health centers, and hospital facilities. People living on and around the Lake use it as a source of drinking water, for bathing and as a toilet. As a consequence, levels of fecal contamination are high, particularly in parts of the lake with stagnant water [16]. This exposure to unsafe water, in turn, leads to a high mortality rate, at 26.1 per 100,000 inhabitants in 2012 [17]. Of the total Malawian population, 59% has no sustainable access to improved sanitation, and 10% do not have safe drinking water sources [17].

### Vaccine procurement

A total of 200,025 doses of Shanchol were shipped from the International Coordinating Group (ICG) emergency stockpile. Vaccine costs were covered by GAVI, the Vaccine Alliance and transported from Kuala Lumpur to Blantyre Expanded Program on Immunization (EPI) storage cold room via Lilongwe.

### Cold chain management

Upon arrival, the vaccines were stored temporarily at the central cold room managed by the Ministry of Health (MoH) in Lilongwe at 2–8 °C. From central level they were transported to the regional cold room in Blantyre. Both cold rooms had sufficient capacity available to absorb the cholera vaccine doses. The cold room in Blantyre is at two hours distance from Machinga, one hour from Zomba and one hour and half from Phalombe. Vaccines were kept at central stores and send at last time possible for distribution. Each vaccination site used one cold box RCW25 and one vaccine carrier for vaccine transportation, with most items borrowed from the EPI program.

### Social mobilization

Before and throughout the vaccination campaign, advocacy and social mobilization activities were conducted to 1) inform the public about the availability of the vaccine against cholera during the campaign; 2) encourage people to get vaccinated against cholera and 3) reinforce good hygiene and sanitation practices. The target areas for social mobilization included gathering places (trading centers, schools, markets, community existing health support groups and fisheries’ organizations). Communication activities were implemented using health education sessions, posters and banners.

### Personnel and training

Training sessions organized all at district level were conducted to train supervisors, vaccinators, and volunteers on vaccine delivery. Sessions were initiated by a two day orientation meeting with the District Executive Committee to gain support from the district administration and partners in the implementation of the campaign. Supervisors were trained simultaneously to ensure consistency between districts. They were subsequently responsible of training vaccination staff and volunteers. Vaccinators were all community based health workers known as health surveillance assistants. Approximately 250 people were trained in the week preceding the vaccination campaign.

### Transportation

A combination of car and boats were used for vaccine delivery to recipients. The transport of vaccine and teams to villages around the Lake Chilwa was done by car and transportation to villages located on the Lake (islands) by boats. For populations in areas not accessible by car or boat, some of the teams moved on foot, bicycle and motorbike for vaccine delivery.

### Data collection

The Global Task Force on Cholera Control (GTFCC) recommended the use of standardized methods for costing assessments of vaccination campaigns. In line with these recommendations, Choltool, a standardized Excel-based cholera cost calculator developed by the International Vaccine Institute, WHO, and DOVE was used for the analysis [18]. The tool quantifies resources required to introduce OCV vaccination campaigns to existing immunization programs. The tool provides estimates of two main cost measures, including the total costs of adding the OCV to specific areas and the cost per fully immunized person. It differentiates financial and economic costs incurred for implementing the OCV vaccination campaign by vaccination related activity (see Table 1).

**Table 1:** Study objectives and type of cost data collected

	Financial costs	Economic costs
Specific objective	To assess the incurred financial costs of implementing the vaccination campaign	To assess the incurred economic costs of the vaccination campaign, including opportunity costs of resources’ utilization
Vaccine purchase	Vaccine costs	Vaccine costs
Vaccine shipment	International transport (freight), clearance insurance, transport and storage	International transport (freight), clearance insurance, transport and storage
Microplanning	Per diems, venue rental, transportation, stationery, printing, fuel and lubricant, catering and communication	Salaried labor, per diems, venue rental, transportation, stationery, printing, fuel and lubricant, catering and communication
Training	Per diems, venue rental, transportation, stationery, printing, fuel and lubricant, catering and communication material, equipment	Salaried labor, per diems, venue rental, transportation, stationery, printing, fuel and lubricant, catering and communication, material, equipment
Sensitization/social mobilization	Per diems, transportation, material, stationery, printing, fuel and lubricant, catering and communication, rental equipment	Salaried labor, volunteer labor, per diems, transportation, stationery, printing, fuel and lubricant, catering and communication, material, equipment
Vaccination rounds	Per diems, transportation, material, stationery, printing, fuel and lubricant, catering and communication, rental equipment, maintenance, operating costs	Salaried labor, volunteer labor, per diems, transportation, material, stationery, printing, fuel and lubricant, catering communication, rental, equipment maintenance, operating costs

Data collection was conducted from February 2016 to March 2017 using various structured questionnaires based on Choltool to estimate the value of resources used to implement the campaign. The data were extracted by reviewing programmatic documents, microplanning, budget, and financial reports basing on actual expenditures and economic costs borne by institutions that supported the implementation of the campaign, including *Agence de Médecine Préventive (AMP)*, *Médecins Sans Frontrières (MSF)*, United Nations Children’s Fund (UNICEF) and WHO.

### Measurement of the vaccination campaign

The vaccination campaign was implemented in two rounds from February 16–22, 2016 and from March 8–15, 2016. The target consisted of 90,000 individuals aged more than one-year-old, including pregnant women. To reach the target, a total of 98 vaccination posts were set up in the selected three districts. Each post, opened daily for a minimum of eight hours, was led by a health surveillance assistant. In total, 53 teams were constituted for the first vaccination round and 56 for the second. Each team consisted of five people, including a vaccinator, a register, a tally person, a logistician, and a social mobiliser. In addition, 23 senior health surveillance assistants were hired to supervise the campaign. Prior to and throughout each round, social mobilization activities were implemented to foster vaccine uptake.

Four structured questionnaires were used to collect costs incurred for conducting each vaccination related activity, including vaccine procurement and shipment, training, microplanning, sensitization, social mobilization and vaccination rounds. Questionnaires, which were derived from Choltool structure, were adapted to data collection at the various levels of campaign implementation and to the Malawian context, including the MoH and various partners which supported the campaign. Financial and economic costs linked to the implementation of the campaign were collected. Data collected included vaccine procurement, shipment of vaccines, personnel and per diems, material and equipment, fuel, lubricant, maintenance, transportation, rental, catering, operating costs, and miscellaneous costs. Capital costs (buildings, cold rooms, vehicles) were excluded from the analysis. Private household costs to receive oral cholera vaccine and costs incurred for monitoring adverse events following immunization (AEFI) were also excluded from this analysis. Details on costs included are described in Table 1. Because the campaign was conducted concurrently with research and treatment activities, efforts were made to disentangle costs of these activities from that of the vaccination.

### Data analysis

The data was entered and analyzed in Choltool [18]. The calculation of the campaign costs followed an ingredient approach to estimate the financial and economic costs of implementing the campaign from the government perspective. The total financial cost was estimated as the sum total of costs incurred for all vaccination related activities described in Table 1. The total cost for each vaccination related activity was obtained by adding up the total costs of all inputs used for that given activity. Each input cost was calculated by multiplying quantities used by the corresponding unit price for recurrent inputs, and annualized before being accounted for some equipment (vaccine carriers, cold boxes). The estimated time costs of each human resource were estimated by multiplying the daily wage by the corresponding time involved. The economic costs captured all resources used, combining financial and opportunity costs for non-marketable resources. Opportunity costs covered some equipment and civil servants’ time. Civil servants’ time costs were accounted for by multiplying the corresponding daily wage of each of these human resources by the corresponding time involved.

The total financial and economic costs of the campaign were reported by vaccination related activities, and further by line input including an estimation of involved international staff costs. This international staff comprised epidemiologists, nurses, logisticians and support staff who contributed to the campaign organization, implementation, supervision as well as vaccine delivery to recipients. The total delivery economic cost was divided by the total number of people receiving the complete vaccine doses to generate the total delivery cost per fully vaccinated person.

Cost data were reported in 2016 US dollars based on OANDA data [19]. To allow comparisons, costs were further converted to international dollars (I$) using the appropriate conversion factor from the International Monetary Fund [20], and estimates reported in supplementary files.

## Results

### Characteristics of the vaccination campaign

Table 2 describes key characteristics of the vaccination campaign. In total, 177,523 doses of vaccine were used, with 108,483 receiving the first dose, and 67,240 persons receiving the complete two doses. About 27% of the first dose was delivered during the second vaccination round. The vaccine coverage with two doses measured with the immunization card and oral reporting was estimated at 58%. Seven mild adverse events following immunization, including vomiting, abdominal cramps, and headache were reported. The proportion of vaccines wasted was very low at, approximately, 1%. The main reasons for wastage included broken vials, empty vials and spillage.

**Table 2:** Descriptive characteristics of the mass oral vaccination campaign

Oral vaccination campaign characteristics	Total
Target population	90,000
Receiving OCV 1st and 2nd dose	67,240
Receiving OCV 1st dose only	108,483
Total number of vaccine doses’ distributed	175,723
Number of vaccine doses’ wasted	1,800
Total number of doses used (distributed plus wasted)	177,523
Two-dose vaccine coverage (%)	58

### Cholera vaccination campaign costs by vaccination related activities and input type

Table 3 reports total financial and economic costs of the vaccination campaign by vaccination related activities. The total economic costs of the vaccination campaign amounted to US$588637. International dollar figures are given in [see Additional file 1]. Economic costs exceeded financial costs by US$108362, with financial costs amounting to US$480275. The total financial and economic costs of vaccination without vaccine totaled US$130319 and US$238681, respectively.

**Table 3:** Distribution of total vaccination costs by activity in 2016 US dollars

	Financial costs		Economic costs	
	2016 US$	Percentage	2016 US$	Percentage
Vaccine procurement and shipment^a^	349,956	72.87	349,956	59.45
Vaccine purchase	331,748	69.08	331,748	56.36
Vaccine shipment, clearance and custom fees	18,208	3.79	18,208	3.09
Vaccine delivery	130,319	27.13	238,681	40.55
Microplanning	11,648	2.42	78,649	13.36
Sensitization	2,865	0.60	9,512	1.62
Training	10,191	2.12	11,097	1.89
Social mobilization	29,377	6.12	29,377	4.99
Vaccination Round 1	34,221	7.12	48,796	8.29
Vaccination Round 2	42,017	8.75	61,250	10.40
Total	480,275	100.00	588,637	100.00

^a^Including wastage

Looking at costs’ distribution by vaccination activities, vaccine procurement and shipment accounted for almost 73% and 59% of total financial and economic costs, respectively, while vaccine delivery costs represented broadly 27% and 41%.

Of the total vaccine delivery economic costs, microplanning was the largest delivery cost component (13%) followed by the vaccination activity itself (8% for round 1 and 10% for round 2). Sensitization activities appeared to be a minor cost component of the delivery cost.

Table 4 reports the breakdown of the total financial and economic costs of vaccination campaign costs by input type in 2016 US$. International dollar figures are given in [see Additional file 2]. The findings show that financial and economic costs for all categories of personnel amounted to US$62386 and US$169313, respectively.

**Table 4:** Distribution of total vaccination costs by input type in 2016 US dollars

	Financial costs		Economic costs	
	2016 US$	Percentage	2016 US$	Percentage
Vaccine procurement and shipment^a^	349,956	72.87	349,956	59.45
Vaccine purchase	328,418	68.38	328,418	55.79
Vaccine shipment, clearance and custom fees	21,538	4.49	21,538	3.66
Vaccine delivery	130,319	27.13	238,681	40.55
Vehicle, fuel, lubricant, and maintenance	32,342	6.73	32,661	5.55
Fuel and ground transportation	25,278	5.26	25,278	4.30
Lubricant and maintenance	124	0.03	124	0.02
Rental (car, boat, etc.)	6,940	1.44	7,259	1.23
Personnel from international partners	0	0.00	90,353	15.35
Salary	0	0.00	62,420	10.61
Per diems	0	0.00	24,326	4.13
International transport and visas	0	0.00	3607	0.61
Personnel, local	62,386	12.99	78,960	13.41
Salary (opportunity cost MoH staff)	0	0.00	16,574	2.81
Per diems (mobilizers, volunteers, local staff, etc.)	62,386	12.99	62,386	10.60
Material	14,483	3.02	15,599	2.65
Banners, T-shirts	12,943	2.70	12,943	2.20
Supplies (printings, plastic bags, etc)	117	0.02	117	0.02
Equipment	1,423	0.30	2,539	0.43
Operating costs	19,753	4.11	19,753	3.36
Operating costs (on-site expenses)	10,299	2.14	10,299	1.75
Communication	9,454	1.97	9,454	1.61
Catering & other expenses	1,355	0.28	1,355	0.23
Beverages, drinks, water, etc	1,092	0.23	1,092	0.19
Miscalleneous	263	0.05	263	0.04
Total costs	480,275	100.00	588,637	100.00

^a^Including wastage

Moreover, the distribution of financial and economic costs shows that, apart from vaccines, incurred total personnel costs were the largest cost component of the total vaccination costs, representing approximately 13% and 29% of total financial and economic costs, respectively. Incurred costs for vehicle, fuel, lubricant, and maintenance came in second place, representing approximately 7% and 6%, respectively, of the total financial and economic costs.

### Cholera vaccine delivery costs by input type

Table 5 shows the breakdown of total vaccine delivery costs by input type. Of the total financial costs for vaccine delivery of US$130319, the incurred total personnel costs represented almost 48%. Considering the economic costs, the incurred total personnel costs accounted for approximately 71% of the total economic costs for vaccine delivery of US$238681. International dollar estimates by input type are shown in [see Additional file 3].

**Table 5:** Distribution of total vaccine delivery costs by input type in 2016 US dollars

	Financial costs		Economic costs	
	2016 US$	Percentage	2016 US$	Percentage
Vehicle, fuel, lubricant, and maintenance	32,342	24.82	32,661	13.68
Fuel and ground transportation	25,278	19.40	25,278	10.59
Lubricant and maintenance	124	0.10	124	0.05
Rental (car, boat, etc.)	6,940	5.32	7,259	3.04
Personnel from international partners	0	0.00	90,353	37.85
Salary	0	0.00	62,420	26.15
Per diems	0	0.00	24,326	10.19
International transport and visas	0	0.00	3,607	1.51
Personnel, local	62,386	47.87	78,960	33.08
Salary (opportunity cost MoH staff)	0	0.00	16,574	6.94
Per diems (mobilizers, volunteers, local staff, etc.)	62,386	47.87	62,386	26.14
Material	14,483	11.11	15,599	6.54
Banners, T-shirts	12,943	9.93	12,943	5.43
Supplies (printings, plastic bags, etc)	117	0.09	117	0.05
Equipment	1,423	1.09	2,539	1.06
Operating costs	19,753	15.16	19,753	8.28
Operating costs (on-site expenses)	10,299	7.90	10,299	4.32
Communication	9,454	7.26	9,454	3.96
Catering & other expenses	1,355	1.04	1,355	0.57
Beverages, drinks, water, etc	1,092	0.84	1,092	0.46
Miscalleneous	263	0.20	263	0.11
Total costs	130,319	100.00	238,681	100.00

Of the latter total, costs incurred for personnel from international partners was the largest vaccine delivery cost item, amounting to US$90353, and accounting for approximately 38% of the total incurred economic costs for vaccine delivery. Costs incurred for local personnel represented 33% of the total economic costs of vaccine delivery. In addition, the economic costs incurred for vehicle, fuel, lubricants, and maintenance represented approximately 14% of the total incurred economic costs for vaccine delivery.

### Unit delivery costs per dose administered and fully vaccinated person

**Fig. 1:**
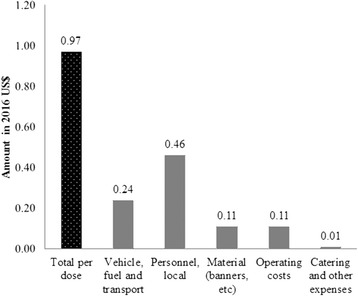
OCV financial delivery cost per dose administered, by activity in 2016 US dollars

Figure 1 shows the distribution of OCV campaign financial costs per dose administered by vaccination related activities. The financial cost per dose administered amounted to US$0.97. The distribution of the unit cost per dose administered by vaccination activities showed that vaccination rounds 1 and 2 were the most important contributors of the unit cost per dose administered.

**Fig. 2:**
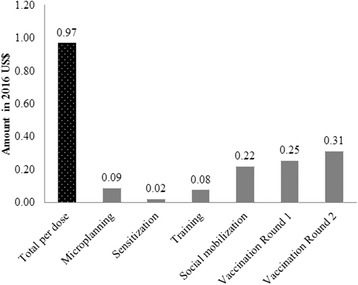
OCV financial delivery cost per dose administered, by input type in 2016 US dollars

The breakdown by input type shows that the incurred local personnel costs constituted the largest contributors of the financial cost per dose administered (Fig. 2).

Table 6 further shows financial and economic unit costs of vaccine delivery per fully vaccinated person. The financial and economic unit costs incurred for delivering the vaccine amounted to US$1.94 and US$3.55, respectively. International dollar estimates by input type are shown in [see Additional file 4]. In total, US$0.93 was spent directly out-of-pocket in personnel costs. In comparison, the unit costs incurred for personnel to deliver the vaccine to recipients amounted to US$2.52 when including the economic costs, with the incurred personnel cost from international partners representing approximately 38% of the incurred unit economic cost per fully vaccinated person.

**Table 6:** Distribution of unit delivery costs per fully vaccinated person by input type in 2016 US dollars

	Financial costs		Economic costs	
	2016 US$	Percentage	2016 US$	Percentage
Vehicle, fuel, lubricant, and maintenance	0.48	24.82	0.49	13.68
Fuel and ground transportation	0.38	19.40	0.38	10.59
Lubricant and maintenance	0.00	0.10	0.00	0.05
Rental (car, boat, etc.)	0.10	5.32	0.11	3.04
Personnel from international partners	0.00	0.00	1.34	37.85
Salary	0.00	0.00	0.93	26.15
Per diems	0.00	0.00	0.36	10.19
International transport and visas	0.00	0.00	0.05	1.51
Personnel, local	0.93	47.87	1.18	33.08
Salary (opportunity cost MoH staff)	0.00	0.00	0.25	6.94
Per diems (mobilizers, volunteers, local staff, etc.)	0.93	47.87	0.93	26.14
Material	0.22	11.11	0.23	6.54
Banners, T-shirts	0.19	9.93	0.19	5.43
Supplies (printings, plastic bags, etc)	0.00	0.09	0.00	0.05
Equipment	0.03	1.09	0.04	1.06
Operating costs	0.29	15.16	0.29	8.28
Operating costs (on-site expenses)	0.15	7.90	0.15	4.32
Communication	0.14	7.26	0.14	3.96
Catering & other expenses	0.02	1.04	0.02	0.57
Beverages, drinks, water, etc	0.02	0.84	0.02	0.46
Miscalleneous	0.00	0.20	0.00	0.11
Total costs	1.94	100.00	3.55	100.00

### Total cholera vaccination costs

With the total financial and economic costs for vaccine procurement and shipment amounting to US$349956 (Table 1), the estimated vaccine procurement and shipment expenditure per fully vaccinated person for this campaign was US$5.20 (349,956/67240). Adding up unit costs of cholera vaccine delivery, total financial and economic costs per fully vaccinated person were US$7.14 and US$8.75, respectively. Total financial and economic costs of vaccination for partially immunized person were US$4.43 and US$5.43, respectively.

## Discussion

This study represents one of the most detailed and comprehensive vaccine delivery costs analysis. With increasing interest in controlling cholera through vaccination, literature estimating the cost of Shanchol™, a low-cost oral cholera vaccine, is growing [21–24]. A limited number of review papers has also been published, reporting cholera vaccination costs in various settings [10, 14]. Some of these studies reported cost estimates disaggregated by inputs [22, 24], while others reported costs by activities [21, 23]. However, none of these studies has detailed enough the costs collected, making it difficult to make comparisons across settings. The comprehensive analysis we propose in this study may therefore orient future studies and contribute to enhancing quality and comparability of costing assessments. Moreover, a number of previous studies only estimated financial costs of vaccination campaigns [21, 23], while this study reported both financial and economic costs. By only reporting financial costs, implications from these previous studies are limited to information on financial needs and do not inform the opportunity costs for the health system (e.g., the costs of MoH staff involvement). This, in turn, limits the potential of studies that only reported financial costs of vaccine delivery in informing cost-effectiveness studies.

The findings showed that total personnel costs accounted to almost 71% of the total economic costs for vaccine delivery and slightly more than 47% of incurred financial costs. The latter result is consistent with the result of a previous review paper, which found that staff salaries accounted for 45% of non-vaccine expenditures [25]. However, this estimate was lower than the 55% estimate given by a previous paper in Bangladesh [24]. The difference in study findings may be due to inflation given that the two studies were from different periods. Moreover, our findings showed that the total incurred economic costs for international partners constituted slightly more than 37% of delivery costs. In their study in Zanzibar, Tanzania, Schaetti et al. reported that international personnel costs accounted for 45% of Dukoral vaccine delivery costs [26]. These two findings suggest that international staff involvement in cholera vaccination campaigns have an important impact on vaccine delivery costs.

The results also showed that cholera vaccine delivery costs accounted for approximately 41% of the total economic costs of the vaccination campaign. Our estimate appeared similar to that of a study which estimated cholera-vaccine delivery costs to account for 42% of total economic costs of vaccination in Bangladesh [24]. It was higher than the study conducted in Zanzibar by Schaetti and al, which found that cholera-vaccine delivery costs represented 32% of total costs of vaccination with Dukoral [26]. Cholera vaccine delivery costs have been estimated at 12% in Odisha, India [23] and 36% of the total vaccination costs in Guinea [21]. The above mentioned three studies may have underestimated the true share of cholera vaccine delivery costs in total vaccination given that economic costs were not accounted for.

The incurred financial cost for vaccine delivery per fully vaccinated person has been estimated at US$1.94. This finding appeared consistent with a previous review paper, which found that incurred financial cost for Shanchol vaccine delivery per fully vaccinated person varied between US$1.14 in India to US$3.05 in South Sudan [14]. However, our estimates may have been pulled up by the fact that external partners were involved in campaign activities implementation on top of local staff and that costs related to international staff salaries and travels may have increased the campaign costs.

Finally, the financial cost incurred per dose administered was US$0.97 of which the incurred personnel cost was US$0.46. This incurred delivery cost per dose administered of US$0.97 was above GAVI’s vaccine operational support for campaigns of US$0.65 per targeted person per vaccination round, intended to cover 80% of MoH-led campaigns’ operational costs [27]. However, our estimate of US$0.97 per dose administered included the incurred international personnel cost which is not covered by GAVI’s support policy. When removing this cost item, GAVI’s support of US$0.65 represented 127% of the financial delivery cost per dose of US$0.51.

This study has limitations. Implementation of vaccination campaigns in many settings have occurred in response to outbreaks. As with previous research [26], some cost data were collected retrospectively. This may have affected our cost estimates. Moreover, salaried labor was estimated retrospectively based on the corresponding daily wage. This may have also contributed to distorting economic costs due to under/overestimation of the time. Capital assets (cold rooms and related equipment, vehicles), particularly from the MoH, were important contributing factors to the vaccination campaign. However, they were not incorporated in our analysis. This also may have contributed to underestimating the true cost of the vaccination campaign. Time and pecuniary costs incurred by household members to travel to vaccination sites and receive free OCV have been shown significant by previous studies [24, 28]. Our study did not analyze private costs incurred by households to receive OCV. Cost estimates may have been higher in comparison of what we presented in this study if the analysis was conducted from the societal perspective, with private costs to receive the vaccine accounted for. Finally, the cost figures we presented in this paper may have also been distorted by the fact that costs incurred for monitoring AEFI were not incorporated.

## Conclusion

Unlike previous costing assessments of cholera vaccination campaign, this study comprehensively presented costs collected and analyzed while clearly distinguishing financial and economic costs as well as activities and inputs. Despite challenges in conducting a detailed analysis of vaccination campaign costs, this type of in-depth analysis should be encouraged for improving understanding and fostering cross-country comparison of costs. The clear description of costs included in this study may therefore be of contribution in this area. The study also showed that international staff expenses are one of the cost drivers that have a substantial impact on cholera vaccine delivery costs.

## Additional files

Additional file 1: Distribution of total vaccination costs by activity in 2016 US dollars and in international dollars (I$). (DOCX 19 kb)

Additional file 2: Distribution of total vaccination costs by input type in 2016 US dollars and in international dollars (I$). (DOCX 23 kb)

Additional file 3: Distribution of total vaccine delivery costs by input type in 2016 US dollars and in international dollars (I$). (DOCX 21 kb)

Additional file 4: Distribution of unit delivery costs per fully vaccinated person by input type in 2016 US$ and international dollars (I$). (DOCX 22 kb)

## Abbreviations

AMP: Agence de Médecine Préventive
Choltool: Cholera Costs Calculator
EPI: Expanded Program on Immunization
GAVI: Global Alliance for Vaccination
GTFCC: Global Task Force on Cholera Control
I$: Global Task Force on Cholera Control
MoH: Ministry of Health
MSF: Médecins Sans Frontières
OCV: Oral Cholera Vaccination
UNICEF: United Nations Children’s Fund
US Dollars: United States Dollars
WHO: World Health Organization
