# Supplementary material for: Delivery cost analysis of a reactive mass cholera vaccination campaign: a case study of Shanchol™ vaccine use in Lake Chilwa, Malawi
Source: BMC Infect Dis. 2017 Dec 19;17:779. doi: 10.1186/s12879-017-2885-8 (PMC5735524; doi:10.1186/s12879-017-2885-8)
Supplement: Supplementary file 1 — Distribution of total vaccination costs by activity in 2016 US dollars and in international dollars (I$). (DOCX 19 kb) [file 12879_2017_2885_MOESM1_ESM.docx]

**Additional file 1: Distribution of total vaccination costs by activity in 2016 US dollars and in international dollars (I$)**

|  | **Financial costs** | | | **Economic costs** | | |
| --- | --- | --- | --- | --- | --- | --- |
|  | **2016 US$** | **I$** | **Percentage** | **2016 US$** | **I$** | **Percentage** |
| **Vaccine procurement and shipment^Ɨ^** | **349 956** | **1 333 332** | **72.87** | **349 956** | **1 333 332** | **59.45** |
| Vaccine purchase | 331 748 | 1 263 960 | 69.08 | 331 748 | 1 263 960 | 56.36 |
| Vaccine shipment, clearance and custom fees | 18 208 | 69 372 | 3.79 | 18 208 | 69 372 | 3.09 |
| **Vaccine delivery** | **130 319** | **496 516** | **27.13** | **238 681** | **909 375** | **40.55** |
| Microplanning | 11 648 | 44 379 | 2.42 | 78 649 | 299 653 | 13.36 |
| Sensitisation | 2 865 | 10 916 | 0.60 | 9 512 | 36 241 | 1.62 |
| Training | 10 191 | 38 828 | 2.12 | 11 097 | 42 280 | 1.89 |
| Social mobilization | 29 377 | 111 926 | 6.12 | 29 377 | 111 926 | 4.99 |
| Vaccination Round 1 | 34 221 | 130 382 | 7.12 | 48 796 | 185 913 | 8.29 |
| Vaccination Round 2 | 42 017 | 160 085 | 8.75 | 61 250 | 233 362 | 10.40 |
| **Total** | **480 275** | **1 829 848** | **100.00** | **588 637** | **2 242 707** | **100.00** |

**^Ɨ^**Including wastage
