# Supplementary material for: Delivery cost analysis of a reactive mass cholera vaccination campaign: a case study of Shanchol™ vaccine use in Lake Chilwa, Malawi
Source: BMC Infect Dis. 2017 Dec 19;17:779. doi: 10.1186/s12879-017-2885-8 (PMC5735524; doi:10.1186/s12879-017-2885-8)
Supplement: Supplementary file 2 — Distribution of total vaccination costs by input type in 2016 US dollars and in international dollars (I$). (DOCX 23 kb) [file 12879_2017_2885_MOESM2_ESM.docx]

**Additional file 2: Distribution of total vaccination costs by input type in 2016 US dollars and in international dollars (I$)**

|  | **Financial costs** | | | **Economic costs** | | |
| --- | --- | --- | --- | --- | --- | --- |
|  | **2016 US$** | **I$** | **Percentage** | **2016 US$** | **I$** | **Percentage** |
| **Vaccine procurement and shipment^Ɨ^** | **349 956** | **1 333 332** | **72.87** | **349 956** | **1 333 332** | **59.45** |
| Vaccine purchase | 328 418 | 1 263 960 | 68.38 | 328 418 | 1 263 960 | 55.79 |
| Vaccine shipment, clearance and custom fees | 21 538 | 69 372 | 4.49 | 21 538 | 69 372 | 3.66 |
| **Vaccine delivery** | **130 319** | **496 516** | **27.13** | **238 681** | **909 375** | **40.55** |
| ***Vehicle, fuel, lubricant, and maintenance*** | **32 342** | **123 222** | **6.73** | **32 661** | **124 439** | **5.55** |
| Fuel and ground transportation | 25 278 | 96 309 | 5.26 | 25 278 | 96 309 | 4.30 |
| Lubricant and maintenance | 124 | 472 | 0.03 | 124 | 472 | 0.02 |
| Rental (car, boat, etc.) | 6 940 | 26 441 | 1.44 | 7 259 | 27 658 | 1.23 |
| ***Personnel from international partners*** | **0** | **0** | **0.00** | **90 353** | **344 243** | **15.35** |
| Salary | 0 | 0 | 0.00 | 62 420 | 237 818 | 10.61 |
| Per diems | 0 | 0 | 0.00 | 24 326 | 92 683 | 4.13 |
| International transport and visas | 0 | 0 | 0.00 | 3 607 | 13 742 | 0.61 |
| ***Personnel, local*** | **62 386** | **237 691** | **12.99** | **78 960** | **300 838** | **13.41** |
| Salary (opportunity cost MoH staff) | 0 | 0 | 0.00 | 16 574 | 63 147 | 2.81 |
| Per diems (mobilizers, volunteers, local staff, etc.) | 62 386 | 237 691 | 12.99 | 62 386 | 237 691 | 10.60 |
| ***Material*** | **14 483** | **55 181** | **3.02** | **15 599** | **59 434** | **2.65** |
| Banners, T-shirts | 12 943 | 49 313 | 2.70 | 12 943 | 49 313 | 2.20 |
| Supplies (printings, plastic bags, etc.) | 117 | 447 | 0.02 | 117 | 447 | 0.02 |
| Equipment | 1 423 | 5 421 | 0.30 | 2 539 | 9 674 | 0.43 |
| ***Operating costs*** | **19 753** | **75 258** | **4.11** | **19 753** | **75 257** | **3.36** |
| Operating costs (on site expenses) | 10 299 | 39 238 | 2.14 | 10 299 | 39 237 | 1.75 |
| Communication | 9 454 | 36 020 | 1.97 | 9 454 | 36 020 | 1.61 |
| ***Catering and other expenses*** | **1 355** | **5 164** | **0.28** | **1 355** | **5 164** | **0.23** |
| Beverages, drinks, water, etc | 1 092 | 4 164 | 0.23 | 1 092 | 4 164 | 0.19 |
| Miscalleneous | 263 | 1 000 | 0.05 | 263 | 1 000 | 0.04 |
| **Total costs** | **480 275** | **1 829 848** | **100.00** | **588 637** | **2 242 707** | **100.00** |

**^Ɨ^**Including wastage
