# Supplementary material for: Delivery cost analysis of a reactive mass cholera vaccination campaign: a case study of Shanchol™ vaccine use in Lake Chilwa, Malawi
Source: BMC Infect Dis. 2017 Dec 19;17:779. doi: 10.1186/s12879-017-2885-8 (PMC5735524; doi:10.1186/s12879-017-2885-8)
Supplement: Supplementary file 3 — Distribution of total vaccine delivery costs by input type in 2016 US dollars and in international dollars (I$). (DOCX 21 kb) [file 12879_2017_2885_MOESM3_ESM.docx]

**Additional file 3: Distribution of total vaccine delivery costs by input type in 2016 US dollars and in international dollars (I$)**

|  | **Financial costs** | | | **Economic costs** | | |
| --- | --- | --- | --- | --- | --- | --- |
|  | **2016 US$** | **I$** | **Percentage** | **2016 US$** | **I$** | **Percentage** |
| **Vehicle, fuel, lubricant, and maintenance** | **32 342** | **123 222** | **24.82** | **32 661** | **124 439** | **13.68** |
| Fuel and ground transportation | 25 278 | 96 309 | 19.40 | 25 278 | 96 309 | 10.59 |
| Lubricant and maintenance | 124 | 472 | 0.10 | 124 | 472 | 0.05 |
| Rental (car, boat, etc.) | 6 940 | 26 441 | 5.32 | 7 259 | 27 658 | 3.04 |
| **Personnel from international partners** | **0** | **0** | **0.00** | **90 353** | **344 243** | **37.85** |
| Salary | 0 | 0 | 0.00 | 62 420 | 237 818 | 26.15 |
| Per diems | 0 | 0 | 0.00 | 24 326 | 92 683 | 10.19 |
| International transport and visas | 0 | 0 | 0.00 | 3 607 | 13 742 | 1.51 |
| **Personnel, local** | **62 386** | **237 691** | **47.87** | **78 960** | **300 838** | **33.08** |
| Salary (opportunity cost MoH staff) | 0 | 0 | 0.00 | 16 574 | 63 147 | 6.94 |
| Per diems (mobilizers, volunteers, local staff, etc.) | 62 386 | 237 691 | 47.87 | 62 386 | 237 691 | 26.14 |
| **Material** | **14 483** | **55 181** | **11.11** | **15 599** | **59 434** | **6.54** |
| Banners, T-shirts | 12 943 | 49 313 | 9.93 | 12 943 | 49 313 | 5.43 |
| Supplies (printings, plastic bags, etc) | 117 | 447 | 0.09 | 117 | 447 | 0.05 |
| Equipment | 1 423 | 5 421 | 1.09 | 2 539 | 9 674 | 1.06 |
| **Operating costs** | **19 753** | **75 258** | **15.16** | **19 753** | **75 257** | **8.28** |
| Operating costs (on-site expenses) | 10 299 | 39 238 | 7.90 | 10 299 | 39 237 | 4.32 |
| Communication | 9 454 | 36 020 | 7.26 | 9 454 | 36 020 | 3.96 |
| **Catering & other expenses** | **1 355** | **5 164** | **1.04** | **1 355** | **5 164** | **0.57** |
| Beverages, drinks, water, etc | 1 092 | 4 164 | 0.84 | 1 092 | 4 164 | 0.46 |
| Miscalleneous | 263 | 1 000 | 0.20 | 263 | 1 000 | 0.11 |
| **Total costs** | **130 319** | **496 516** | **100.00** | **238 681** | **909 375** | **100.00** |
