# Supplementary material for: Delivery cost analysis of a reactive mass cholera vaccination campaign: a case study of Shanchol™ vaccine use in Lake Chilwa, Malawi
Source: BMC Infect Dis. 2017 Dec 19;17:779. doi: 10.1186/s12879-017-2885-8 (PMC5735524; doi:10.1186/s12879-017-2885-8)
Supplement: Supplementary file 4 — Distribution of unit delivery costs per fully vaccinated person by input type in 2016 US$ and international dollars (I$). (DOCX 22 kb) [file 12879_2017_2885_MOESM4_ESM.docx]

**Additional file 4:** **Distribution of unit delivery** **costs per fully vaccinated person by input type in 2016 US$ and international dollars (I$)**

|  | **Financial costs** | | | **Economic costs** | | |
| --- | --- | --- | --- | --- | --- | --- |
|  | **2016 US$** | **I$** | **Percentage** | **2016 US$** | **I$** | **Percentage** |
| **Vehicle, fuel, lubricant, and maintenance** | **0.48** | **1.83** | **24.82** | **0.49** | **1.85** | **13.68** |
| Fuel and ground transportation | 0.38 | 1.43 | 19.40 | 0.38 | 1.43 | 10.59 |
| Lubricant and maintenance | 0.00 | 0.01 | 0.10 | 0.00 | 0.01 | 0.05 |
| Rental (car, boat, etc.) | 0.10 | 0.39 | 5.32 | 0.11 | 0.41 | 3.04 |
| **Personnel from international partners** | **0.00** | **0.00** | **0.00** | **1.34** | **5.12** | **37.85** |
| Salary | 0.00 | 0.00 | 0.00 | 0.93 | 3.54 | 26.15 |
| Per diems | 0.00 | 0.00 | 0.00 | 0.36 | 1.38 | 10.19 |
| International transport and visas | 0.00 | 0.00 | 0.00 | 0.05 | 0.20 | 1.51 |
| **Personnel, local** | **0.93** | **3.53** | **47.87** | **1.18** | **4.47** | **33.08** |
| Salary (opportunity cost MoH staff) | 0.00 | 0.00 | 0.00 | 0.25 | 0.94 | 6.94 |
| Per diems (mobilizers, volunteers, local staff, etc.) | 0.93 | 3.53 | 47.87 | 0.93 | 3.53 | 26.14 |
| **Material** | **0.22** | **0.82** | **11.11** | **0.23** | **0.88** | **6.54** |
| Banners, T-shirts | 0.19 | 0.73 | 9.93 | 0.19 | 0.73 | 5.43 |
| Supplies (printings, plastic bags, etc) | 0.00 | 0.01 | 0.09 | 0.00 | 0.01 | 0.05 |
| Equipment | 0.03 | 0.08 | 1.09 | 0.04 | 0.14 | 1.06 |
| **Operating costs** | **0.29** | **1.12** | **15.16** | **0.29** | **1.12** | **8.28** |
| Operating costs (on-site expenses) | 0.15 | 0.58 | 7.90 | 0.15 | 0.58 | 4.32 |
| Communication | 0.14 | 0.54 | 7.26 | 0.14 | 0.54 | 3.96 |
| **Catering & other expenses** | **0.02** | **0.08** | **1.04** | **0.02** | **0.08** | **0.57** |
| Beverages, drinks, water, etc | 0.02 | 0.06 | 0.84 | 0.02 | 0.06 | 0.46 |
| Miscalleneous | 0.00 | 0.02 | 0.20 | 0.00 | 0.02 | 0.11 |
| **Total costs** | **1.94** | **7.38** | **100.00** | **3.55** | **13.52** | **100.00** |
